# MDM2’s dual mRNA binding domains co-ordinate its oncogenic and tumour suppressor activities

**DOI:** 10.1093/nar/gkaa431

**Published:** 2020-05-26

**Authors:** Sivakumar Vadivel Gnanasundram, Laurence Malbert-Colas, Sa Chen, Leila Fusée, Chrysoula Daskalogianni, Petr Muller, Norman Salomao, Robin Fåhraeus

**Affiliations:** Inserm UMRS1131, Institut de Génétique Moléculaire, Université Paris 7, Hôpital St. Louis, F-75010 Paris, France; Inserm UMRS1131, Institut de Génétique Moléculaire, Université Paris 7, Hôpital St. Louis, F-75010 Paris, France; Department of Medical Biosciences, Building 6M, Umeå University, 901 85 Umeå, Sweden; Inserm UMRS1131, Institut de Génétique Moléculaire, Université Paris 7, Hôpital St. Louis, F-75010 Paris, France; Inserm UMRS1131, Institut de Génétique Moléculaire, Université Paris 7, Hôpital St. Louis, F-75010 Paris, France; RECAMO, Masaryk Memorial Cancer Institute, Zlutykopec 7, 65653 Brno, Czech Republic; Inserm UMRS1131, Institut de Génétique Moléculaire, Université Paris 7, Hôpital St. Louis, F-75010 Paris, France; Inserm UMRS1131, Institut de Génétique Moléculaire, Université Paris 7, Hôpital St. Louis, F-75010 Paris, France; Department of Medical Biosciences, Building 6M, Umeå University, 901 85 Umeå, Sweden; RECAMO, Masaryk Memorial Cancer Institute, Zlutykopec 7, 65653 Brno, Czech Republic; ICCVS, University of Gdańsk, Science, ul. WitaStwosza 63, 80-308 Gdańsk, Poland

## Abstract

Cell growth requires a high level of protein synthesis and oncogenic pathways stimulate cell proliferation and ribosome biogenesis. Less is known about how cells respond to dysfunctional mRNA translation and how this feeds back into growth regulatory pathways. The Epstein-Barr virus (EBV)-encoded EBNA1 causes mRNA translation stress *in cis* that activates PI3Kδ. This leads to the stabilization of MDM2, induces MDM2’s binding to the *E2F1* mRNA and promotes *E2F1* translation. The MDM2 serine 166 regulates the interaction with the *E2F1* mRNA and deletion of MDM2 C-terminal RING domain results in a constitutive *E2F1* mRNA binding. Phosphorylation on serine 395 following DNA damage instead regulates *p53* mRNA binding to its RING domain and prevents the *E2F1* mRNA interaction. The p14Arf tumour suppressor binds MDM2 and in addition to preventing degradation of the p53 protein it also prevents the *E2F1* mRNA interaction. The data illustrate how two MDM2 domains selectively bind specific mRNAs in response to cellular conditions to promote, or suppress, cell growth and how p14Arf coordinates MDM2’s activity towards p53 and E2F1. The data also show how EBV via *EBNA1*-induced mRNA translation stress targets the E2F1 and the MDM2 - p53 pathway.

## INTRODUCTION

Oncogenic viruses like Simian virus 40 (SV40), human papilloma virus (HPV) and adenovirus target the pRb-E2F and the p53 pathways and stimulate cell proliferation (1). The Epstein-Barr virus (EBV) was the first human oncogenic virus to be discovered and is associated with approximately 2% of human cancers but its oncogenic mechanisms are not fully understood (2). The EBV-encoded EBNA1 is essential for viral replication and is expressed in all virus-carrying cells (3). We have previously shown that EBNA1 suppresses its own translation *in cis* to minimize the production of antigenic peptides for the major histocompatibility complex (MHC) class I pathway. This causes mRNA translation stress which leads to an increase in cell proliferation and ribosomal biogenesis by stimulating E2F1 synthesis and c-myc expression in a PI3Kδ-dependent fashion (4–9). Suppressing PI3Kδ reduces E2F1 expression also in non-EBV infected tumour cells, demonstrating that this pathway is also active in rapidly proliferating cells. Treating transgenic EBNA1-induced B cell lymphomas with the PI3Kδ kinase inhibitor CAL-101 (Idelalisib) reduce the levels of E2F1 and c-myc and causes cell death (8). Hence, EBNA1 mediates its oncogenic activity by suppressing its own synthesis, which explains why two transgenic animal models show an inverse phenotype between EBNA1 protein expression and tumour phenotype (10,11).

Both loss and gain of PI3Kδ function has been linked to immune deficiency syndromes and to affect T and B cell populations but it is also detected in non-immune cells. Patients with gain of function mutations show activated PI3Kδ syndrome (APDS or PASLI) and have impaired B cell maturation and increased risk of developing B cell lymphoma (12).

Murine double minute 2 (MDM2 ((HDM2in human)) is a multifunctional intrinsically disordered protein that is amplified in approximately 10% of cancers including sarcomas, lymphomas, and B-cell lymphocytic leukemia (13–17). MDM2 is a key regulator of the p53 tumour suppressor and mice lacking MDM2 die early during embryogenesis in a p53-dependent fashion (18). Under normal conditions, MDM2 binds the N-terminus of p53 and blocks its transcriptional activity, changes its subcellular localization and promotes p53 ubiquitination (19–23). The p14Arf tumour suppressor is induced by E2F1 following oncogenic stress and interacts with the core domain of MDM2 to prevent p53 degradation (24–30). During DNA damage, MDM2 is phosphorylated at serine 395 by the ATM kinase and this switches MDM2 to become a positive regulator of p53 by binding the *p53* mRNA and stimulate p53 synthesis (31–34). In addition to its control of p53, MDM2 also interacts with a large number of cellular factors regulating cell growth and proliferation, including ribosomal factors such as RPL5, RPL11, RPL23 and the 5S RNP complex (35,36). MDM2 has been reported to induce E2F1 levels, directly or via pRb degradation, and to promote cell proliferation and oncogenesis (37–39). However, a negative regulation of E2F1 by MDM2 has also been reported (40–43).

Here we show that EBNA1-induced mRNA translation stress stimulates the MDM2 - *E2F1* mRNA interaction and promotes *E2F1* mRNA translation. This reveals a feedback pathway whereby cells sense dysfunctional mRNA translation and via PI3Kδ and MDM2 induces the expression of E2F1 and c-myc to restore ribosomal biogenesis to promote cell growth. The data also illustrate how MDM2, depending on cellular conditions and via different RNA binding domains, binds the *p53* or *E2F1* mRNAs and thereby acts as an oncogene or tumour suppressor and how p14Arf manages MDM2’s activity towards p53 and E2F1.

## MATERIALS AND METHODS

### Cell culture, transfection and drug treatments

Experiments were performed mostly using H1299 cells (non-small-cell lung carcinoma human cell line) [NCI-H1299 (ATCC® CRL5803™)], unless stated otherwise. Other cell lines used were MDM2/MDMX double KO H1299 cell line (44), A549 cells (Adenocarcinomic human alveolar basal cell line), U2OS cells (Human bone osteosarcoma epithelial Cell line), SAOS-2 cells (Human bone osteosarcoma cell line), A375 (p53WT), A375 (p53KO) (Human melanoma cell line) and Raji cells (type III latent Burkitt's Lymphoma). Cell lines were cultured in RPMI 1640 medium or in DMEM–Dulbecco's Modified Eagle Medium (for U2OS, A375 and A549 cell lines) supplemented with 10% fetal bovine serum, 2 mM L-glutamine, 100 U/ml penicillin, 100 μg/ml streptomycin (Invitrogen) and 5 μg/ml Plasmocin prophylactic (Invivogen). Cell lines were routinely checked for mycoplasma contamination using PlasmoTest kit (Invivogen). Drugs: MG132 (474790-5, Calbiochem), PI3Kδ inhibitor CAL-101 (S2226, Selleck Chemicals), PI-3065 (S7623, Selleck Chemicals), AKT kinase inhibitor Ipatasertib (GDC-0068) (SES22808, Selleck Chemicals), Doxorubicin (Sigma-Aldrich), Cycloheximide (C4859, Sigma-Aldrich).

### Plasmid constructs and siRNAs

Plasmid constructs were created using the eukaryotic expression vector pcDNA3. The plasmid constructs pcDNA3-E2F1, pcDNA3-E2F1Δ324, pcDNA3-E2F1Δ432, pcDNA3-EBNA1, pcDNA3-EBNA1ΔGAr, pcDNA3-p53, pcDNA3-MDM2, pcDNA3-HDM2, pcDNA3-p14ARF, pET28-MDM2 were all described previously (8,34,45). Using site directed mutagenesis pcDNA3-MDM2-S166D, pcDNA3-MDM2-S166A, pcDNA3-MDM2-S186D, pcDNA3-MDM2-S186A, pET28-MDM2-S166D, pET28-MDM2-S166A were created. For silencing MDM2, the FlexiTube GeneSolution siRNAs against *mdm2* (GS4193, Qiagen) were used. For transfection of plasmid DNA and siRNAs, Genejuice (EMD chemicals) and INTERFERin (polyplus transfection) reagents were used respectively according to the manufacturer's instructions.

### Western blot analysis

Cells were lysed using BC200 lysis buffer (200 mM NaCl, 0.2% NP-40, 10% (v/v) glycerol, 1.0 mM dithiothreitol (DTT), 1.0 mM EDTA, and 25 mM Tris-HCl, pH 7.8) containing 1% (v/v) eukaryotic protease inhibitor cocktail (Calbiochem). Equal protein amounts were loaded and resolved in 10% Bis–Tris Plus Gels (Thermo Fisher), transferred on BioTrace NT pure nitrocellulose blotting membrane (PALL Corporation) and blocked with 5% non-fat dry milk in Tris-buffered saline pH 7.6 containing 0.1% Tween-20. Proteins were then probed with corresponding antibodies; anti-E2F1 rabbit pAbs [1:1000] (C-20, Santa Cruz), anti-MDM2 mouse mAbs [1:500] (4B2-Recamo), anti-p53 rabbit pAbs [1:1000] (CM-1-Recamo), anti-p14ARF mouse mAbs [1:2000] (sc-53639, Santacruz), anti-phospho-MDM2 (Ser166)[1:500] (3521S, Cell Signaling Technology) and anti-Actin mouse pAbs [1:2000] (AC-15, Sigma-Aldrich).

### Metabolic pulse labelling

Pulse labelling was performed as described previously (8). Briefly, 24 h post transfection, cells were cultured for 1 h in Dulbecco's modified Eagle's starvation medium (Sigma-Aldrich) (without methionine, cysteine and L-glutamine, supplemented with 2% dialysed fetal bovine serum together with 20 μM of proteasome inhibitor MG132. Cells were metabolically labelled for 1 h with 45 μCi/ml of EasyTag Express ^35^S-methionine Protein Labelling Mix (Perkin-Elmer). E2F1 and MDM2 proteins were then immunoprecipitated with corresponding antibodies (E2F1 mouse mAbs (321400, Life Technologies), anti-MDM2 mouse mAbs (4B2-Recamo) using Dynabeads™ Protein G Immunoprecipitation Kit (10007D, ThermoFischer) and eluted proteins were resolved in 4–12% Bis–Tris Plus Gels and visualized on autoradiograph.

### RNA extraction, RT-PCR and qRT-PCR

H1299 cells were plated in six-well plates and transfected with the indicated constructs. 48 h post transfection, cells were washed with cold PBS and total RNA was purified using the RNeasy Mini Kit and on-column DNase treatment (74104, Qiagen) following the manufacturer's protocol. RT was carried out using Moloney Murine Leukaemia Virus Reverse Transcriptase and random hexamers or oligo(dT) primers (Invitrogen). RT-qPCR was performed on StepOne real-time PCR system (Applied Biosystems) using Perfecta SYBR Green FastMix, ROX (Quanta Biosciences) (See Supplementary Table S1 for target primer sequences).

### Proximity ligation assays

H1299 cells were plated on 12-mm-diameter coverslips in 24-well plates, and transfected with the indicated constructs or vector control (EV). 24 h post-transfection cells were fixed with 4% paraformaldehyde (PFA) for 20 min, permeabilized with PBS 0.4% Triton X-100, 0.05% CHAPS for 10 min at room temperature and saturated with PBS 3% BSA for 30 min. Samples were hybridized overnight at 37 °C with *E2F1* RNA probe (5′-TTCTCCTCCTCAGAAGTGACCTCCTGAAAA-3′) conjugated to biotin in the 3′ end. Afterwards, samples were saturated with PBS 3% BSA, 0.1% saponine and incubated for 2 h with anti-MDM2 mouse mAbs (4B2) and anti-biotin rabbit mAbs (5597, Cell Signaling technology) primary antibodies diluted in blocking solution. The proximity ligation assay (PLA) was carried out using the Duolink PLA in situ kit (Sigma) following the manufacturer's protocol. Cover slips were mounted in the DAKO medium and images were acquired using Carl-Zeiss Axiovert inverted microscope.

### Ex vivo and in vitro RNA co-immunoprecipitation

*Ex vivo* MDM2 RNA co-immunoprecipitation (RNA co-IP) was carried out using Dynabeads™ Protein G Immunoprecipitation Kit (ThermoFischer) following the manufacturer's instructions, with anti-MDM2 mouse antibody (4B2). Briefly, 48 h post-transfection cells were lysed using IP lysis buffer (100 mM KCl, 50 mM HEPES-KOH, 5 mM MgCl2, 1 mM DTT, 0.5% NP-40, Proteasome inhibitor cocktail). Cleared lysate was used for MDM2-IP with Protein G dynabeads. RNA was purified from input and IP- elute samples using TRIzol (Life Technologies) and analyzed by RT-qPCR for *E2F1* mRNA enrichment. Percentage of enrichment of *E2F1* mRNA from the input normalized with actin was plotted for both EV and GAr.

*In vitro* RNA co-IP was carried out as described (34,46) using monoclonal MDM2 antibody (4B2). Briefly, 1 μg of total RNA from transfected cells were co-incubated under agitation with 100 ng of recombinant MDM2 in binding buffer (50 mM Tris pH 7.5, 150 mM NaCl, 0.02 mg/ml yeast tRNA, 0.2 mg/ml BSA) at 4°C. After incubation, MDM2 – RNA complexes were pulled down using protein G-coated sepharose beads (Sigma-Aldrich) according to standard conditions and purified using the TRIzol (Life Technologies). Precipitated RNAs were analyzed by RT-qPCR for mRNA enrichment using primers listed in Supplementary Table S1. Fold enrichment of mRNA levels between cells expressing EV and GAr was plotted in the graph.

For the *in vitro* mRNA chaperoning assay, *in vitro* transcription of *E2F1* and *p53* mRNAs were carried out in the presence, or absence, of MDM2 S166D protein. The mRNAs were then isolated and proteins removed before the mRNAs were incubated with recombinant MDM2-WT protein followed by i*n vitro* RNA co-IP to determine the relative interaction between protein and RNA (as described above).

### Polysome profiling

5–50% (w/v) linear sucrose gradients were freshly casted on SW41 ultracentrifuge tubes (Beckmann) using the Gradient master (BioComp instruments) following the manufacturer's instructions. 48 h post transfection, H1299 cells (with 80% confluency) were treated with cycloheximide 100 μg/ml for 5 min at 37°C and washed twice with 1 × PBS (Dulbecco modified PBS, GIBCO) containing cycloheximide 100 μg/ml. Cells were lysed with polysome lysis buffer (100 mM KCl, 50 mM HEPES–KOH, 5 mM MgCl2, 0.1% NP-40, 1 mM DTT, cycloheximide 100 μg/ml, pH 7.4) and clear lysate was loaded on a sucrose gradient and centrifuged at 222 228 × g for 2 h at 4°C in a SW41 rotor. Samples were fractionated using Foxy R1 fraction collector (Teledyne ISCO) at 0.5 min intervals. Collected fractions were then pooled accordingly (free pool, 40S/60S, monosome and polysome fractions), concentrated using Amicon® Ultra-15 Centrifugal Filter Units (Merckmillipore) and subjected to Western and RT-qPCR analysis. RNA purifications from fractions were performed using ethanol precipitation combined with RNeasy Mini Kit (Qiagen). RT and qPCR were performed as described above using primers described in Supplementary Table S1. The ratio of the fold enrichment of *E2F1* mRNA levels in the stress induced cells (GAr) to the normal cells (EV) were plotted, actin levels were used for the normalization.

### Statistical analysis

Statistical significance was analyzed by comparing data with corresponding reference points using two-tailed *t* tests (*P < 0.05; **P < 0.01; ***P < 0.001; ns, not significant). All statistical assessments were performed using the Microsoft excel program.

## Results

### MDM2 enhances E2F1 synthesis following EBNA1-induced translation stress

**Figure 1.**
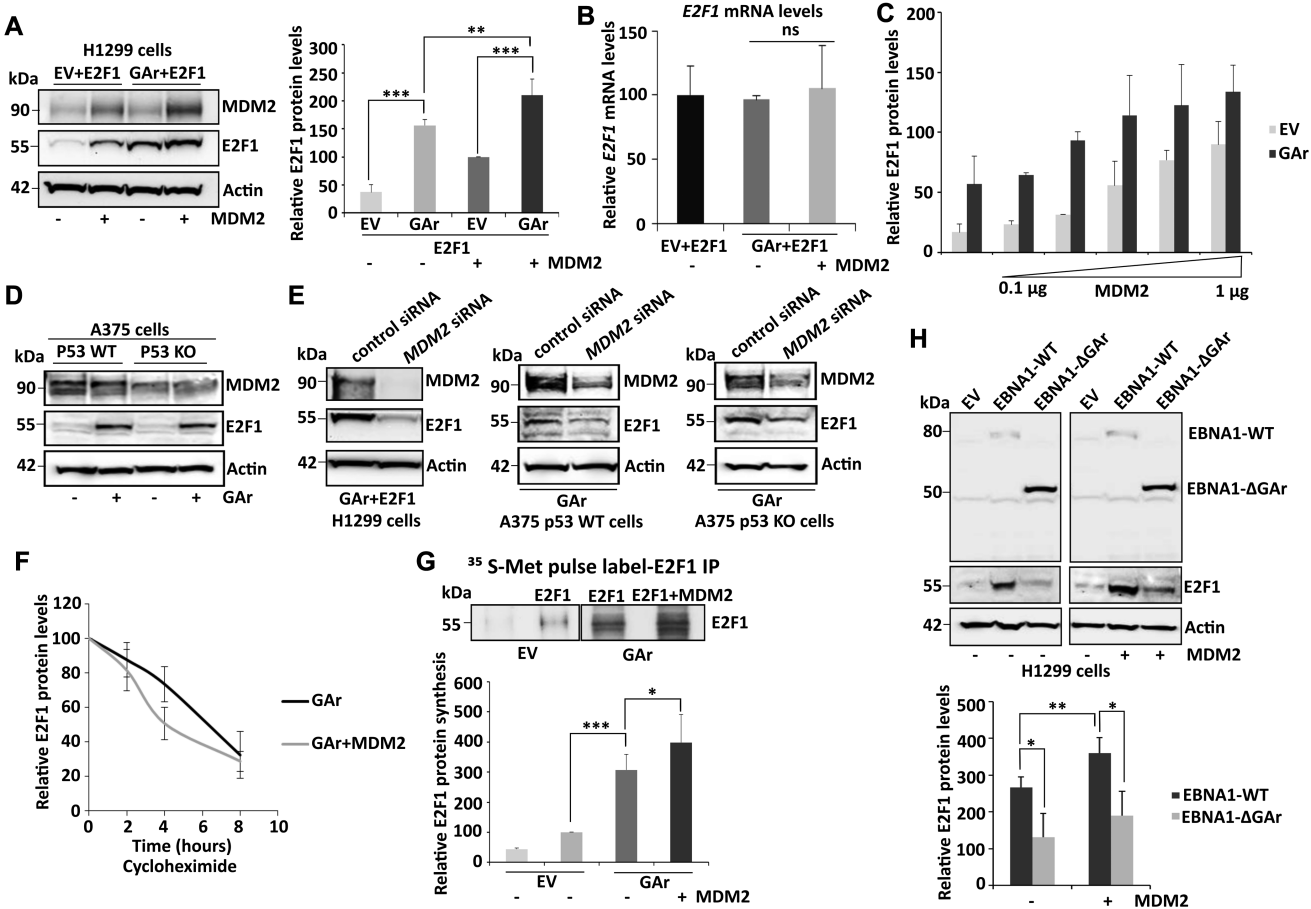
MDM2 regulates E2F1 synthesis during mRNA translation stress. (**A**) Western blot (WB) showing E2F1 levels in H1299 cells expressing MDM2 and E2F1 in normal (EV) conditions and during mRNA translation stress induced by the EBNA1-derived GAr. Panel (right) shows the quantification of E2F1 protein levels from three independent experiments. Actin levels were used for normalization. (**B**) The *E2F1* mRNA levels were analyzed by RT-qPCR under conditions as indicated in (A). (**C**) Graph shows quantification of the E2F1 protein levels in H1299 cells expressing E2F1 under normal (EV) and mRNA translation stress (GAr) conditions with increasing amount of MDM2 (see also Supplementary Figure S2A). (**D**) WB showing the levels of endogenous E2F1 in A375 cells (p53 WT) and in A375 cells lacking p53 (p53 KO) following mRNA translation stress conditions. (**E**) The levels of E2F1 in indicated cells following treatment with control siRNA and siRNAs against MDM2. (**F**) Quantification of E2F1 protein levels at indicated time points after cycloheximide treatment in H1299 cells expressing the GAr, with, or without, MDM2 (see also Supplementary Figure S1E) (**G**) The rate of newly synthesized E2F1 protein levels is shown by autoradiograph of ^35^S-Met Pulse labelled H1299 cells followed by E2F1 immunoprecipitation in the presence of proteasome inhibitor MG132. Graph below shows quantification and relative values of three independent experiments. (**H**) WB shows the levels of E2F1 in H1299 cells expressing EBNA1-WT and EBNA1 lacking the GAr (EBNA1-ΔGAr) in the presence, or absence, of MDM2. The graph below shows quantification of data from three independent experiments. Western blots represent one of three independent experiments and actin was used as a loading control. Statistical significance was calculated using t tests (***P < 0.001, **P < 0.01 and *P < 0.05).

We have previously shown that when the gly-ala repeat (GAr) of the EBNA1 is fused to the 5′ coding sequence of mRNAs it causes translation stress *in cis* and this stimulates cell proliferation by promoting E2F1 synthesis in a PI3Kδ-dependent fashion (8). In this study, we have taken advantage of the unique features of the GAr that allows us to control mRNA translation stress without treating cells with general inhibitors of protein synthesis to uncover the molecular mechanisms of E2F1 induction. MDM2 is known to both stimulate and suppress E2F1 expression that shows similarities to its condition-dependent activity towards p53 that is regulated by its binding to the p53 protein or to the *p53* mRNA (32,34). We therefore examined MDM2’s activity towards E2F1 during GAr-induced mRNA translation stress in p53 null H1299 cells and we observed an increase in E2F1 levels following expression of the GAr, that was further enhanced by the expression of MDM2 (similar data was observed with the human MDM2 (HDM2), without significant changes in the *E2F1* mRNA levels (Figure 1A, B and Supplementary Figure S1A). The fact that MDM2 induced E2F1 also in the absence of the GAr is in line with the previous observation that the PI3Kδ translation stress pathway is active also in non-GAr expressing cells (8) (see also further below). We observed a similar induction of E2F1 levels upon overexpression of MDM2 in A549 (p53 wild type adenocarcinoma) and SAOS-2 (p53 null osteosarcoma) cells (Supplementary Figures S1B, S1C and S1D). Furthermore, a gradual increase in MDM2 levels during translation stress resulted in a corresponding increase in E2F1 protein levels. However, doubling the amount of transfected MDM2 from 1 to 2 μg resulted in a decrease in E2F1 expression, indicative of MDM2 also having the capacity to target E2F1 for degradation (Figure 1C and Supplementary Figures S2A and S2B). H1299 cells have low levels of MDM2 and E2F1 and to determine the effect of translation stress on endogenous E2F1 levels we expressed the GAr in A375 cells, or in A375 cells lacking p53 (A375 KO). This resulted in an GAr-mediated induction of endogenous E2F1 levels in both cells, showing that the effect is not p53-dependent (Figure 1D). When H1299 and the two A375 cell lines were treated with siRNA against MDM2, E2F1 protein levels were significantly reduced and the translation stress-induced expression was abrogated, confirming the requirement of MDM2 for E2F1 expression under these conditions (Figure 1E). We next treated cells with the protein synthesis inhibitor cycloheximide (CHX) and we observed that overexpressing MDM2 had no effect on E2F1 turnover rate over 8 hours, indicating that the effect of MDM2 on the increase in E2F1 expression following translation stress is not at the level of protein stability (Figure 1F and Supplementary Figure S1E). ^35^S-Met metabolic pulse label in the presence of 20 μM of the proteasome inhibitor MG132, showed that mRNA translation stress induces newly synthesized E2F1 and this is further increased in the presence of MDM2 (Figure 1G). The induction of E2F1 by EBNA1, and not by an EBNA1 that lacks the GAr (EBNA1-ΔGAr), confirms that the translation stress-dependent induction of E2F1 requires the GAr sequence of EBNA1 (Figure 1H). Altogether, these results show that MDM2 is required for mRNA translation stress-induced synthesis of E2F1.

### MDM2 interacts with the E2F1 mRNA during translation stress

**Figure 2.**
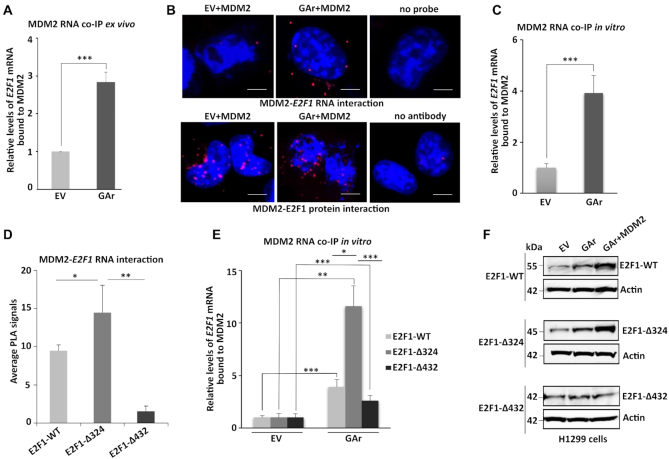
MDM2 interacts with the *E2F1* mRNA during GAr-induced mRNA translation stress. (**A**) Graph shows the fold enrichment of *E2F1* mRNA co-immunoprecipitated with MDM2 (RNA co-IP) from H1299 cell lysates under normal (EV) and mRNA translation stress (GAr) conditions. (**B**) Proximity ligation assay (PLA) shows the MDM2 protein – *E2F1* mRNA interactions (upper panel, red dots) and MDM2 – E2F1 protein-protein interactions (lower panel, red dots) *in situ* under normal and translation stress conditions in H1299 cells. (**C**) The graph shows *E2F1* mRNA bound to recombinant MDM2. Total mRNA was isolated from H1299 cell lysates expressing EV or the GAr and used for *in vitro* RNA co-IP with recombinant purified MDM2 protein followed by RT-qPCR against *E2F1* mRNA. (**D**) PLA quantification (average number of dots per cell) of *in situ* MDM2 – *E2F1* mRNA interactions using indicated E2F1-WT, E2F1-Δ324 and E2F1-Δ432 constructs in H1299 cells (see also Supplementary Figure S3). (**E**) Graph shows relative GAr-dependent enrichment of indicated *E2F1* mRNAs bound to recombinant MDM2. (**F**) WB showing protein levels from indicated E2F1 constructs in H1299 cells expressing the GAr with, or without, over expressing MDM2. Actin was used as a loading control and the WB data shows one of three representative experiments. Statistical significance was calculated using t tests (***P < 0.001, **P < 0.01 and *P < 0.05) of three independent experiments. Scale bars 10 μm.

To understand MDM2’s stimulatory role towards E2F1 during mRNA stress conditions, we examined the interaction between MDM2 and the *E2F1* mRNA by immunoprecipitating MDM2 followed by RT-qPCR against the *E2F1* mRNA (RNA co-IP). The presence of the GAr resulted in an approximately three-fold enrichment of *E2F1* mRNA bound to MDM2 (Figure 2A). We also performed *in situ* proximity ligation assay (PLA) against the MDM2 – *E2F1* mRNA interaction using antibodies against MDM2 together with the combination of biotinylated *E2F1* RNA probes and anti-biotin antibodies. We also carried out PLA against the MDM2 – E2F1 protein-protein interaction. Under normal conditions we detected on average one to two MDM2 – *E2F1* mRNA and about 20 MDM2 – E2F1 protein-protein interactions per cell. The expression of the GAr resulted in an approximately 10-fold increase of MDM2 – *E2F1* mRNA interactions in both the nuclear and cytoplasmic compartments, while the number of protein-protein interactions did not change significantly (Figure 2B). We next carried out *in-vitro* RNA co-IP using recombinant purified MDM2 proteins and total RNAs isolated from control (EV) and translation stress-induced cells (GAr), followed by RT-qPCR against the *E2F1* mRNA. Interestingly, there was an increase in the binding of *E2F1* mRNA to recombinant MDM2 proteins from translation stress-induced cells, as compared to control (Figure 2C). When we performed the PLA using a series of truncated *E2F1* mRNA constructs (8), we observed a significant increase in the *E2F1* mRNA – MDM2 interaction following deletion of the first 324 nucleotides (Δ324) of the coding sequence of E2F1. Further deletion to +432 (Δ432) resulted in a significant loss of interaction, indicating the minimal MDM2-binding region lies between +324 to +432 (Figure 2D and Supplementary Figure S3). In line with this, when we carried out RNA co-IP we observed the strongest MDM2 – *E2F1* mRNA interaction using the E2F1-Δ324 construct (Figure 2E). We also observed the highest MDM2-mediated induction of protein expression using the truncated E2F1-Δ324 construct under stress conditions, while we did not observe any induction using the E2F1-Δ432 (Figure 2F). These results show that the *E2F1* mRNA is made accessible to MDM2 following translation stress and indicate a direct correlation between the binding of MDM2 to the *E2F1* mRNA and MDM2-mediated induction of E2F1 expression.

### The RING domain of MDM2 and serine 166 regulate translation stress-induced binding to the E2F1 mRNA

**Figure 3.**
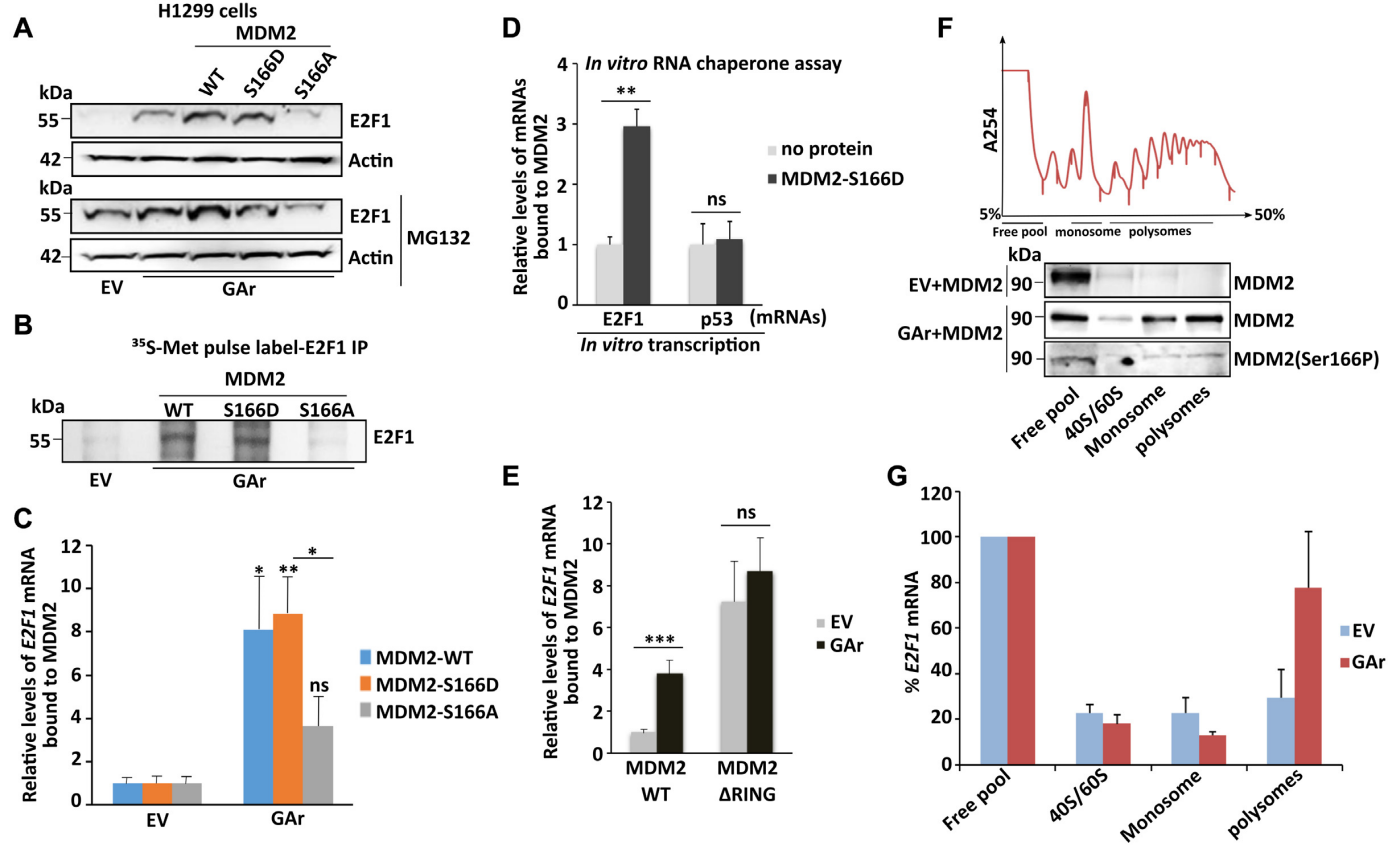
MDM2 phosphorylation at serine 166 and the RING domain control MDM2 interaction with the *E2F1* mRNA during translation stress. (**A**) WB showing E2F1 levels in H1299 cells expressing MDM2-WT, MDM2-S166D and the MDM2-S166A under normal and translation stress conditions in the presence (lower), or absence (upper) of the proteasome inhibitor MG132 (20 μM for 2 h). (**B**) Autoradiograph of ^35^S-Met metabolic pulse labeling in the presence of MG132 (20 μM) followed by E2F1 immunoprecipitation shows the rate of newly synthesized E2F1 proteins in the presence of indicated MDM2 constructs in H1299 cells. (**C**) H1299 cells expressing the MDM2-WT, MDM2-166D or the MDM2-166A constructs. Total RNA was isolated from cells under normal or translation stress (GAr) conditions and the graph shows the relative amount of *E2F1* mRNA bound to recombinant MDM2-WT, MDM2-S166D and MDM2-S166A proteins, respectively. (D) Graph shows the binding of recombinant MDM2 protein to *E2F1* and *p53* mRNAs transcribed *in vitro* in the presence, or absence, of MDM2-S166D protein. Following RNA synthesis, the mRNAs were isolated and proteins removed and the relative amount bound to recombinant MDM2 protein was estimated. (**E**) The relative levels of *E2F1* mRNA bound to recombinant MDM2-WT and to an MDM2 lacking the C-terminal RING domain (MDM2-ΔRING). Total RNA was isolated from H1299 cells lysates under indicated conditions. (**F**) Polysome profiling followed by WB analysis of pooled fractions under normal and translation stress conditions using MDM2 (4B2) and phospho-MDM2 (Ser166P) antibodies. (**G**) RT-qPCR analysis of *E2F1* mRNA from corresponding fractions (F), enrichment of *E2F1* mRNA in normal (EV) and translation stress conditions were plotted (see also Supplementary Figure S4D). Values were normalized with actin levels and are representative of three independent experiments. Statistical significance was calculated using t tests (***P < 0.001, **P < 0.01 and *P < 0.05).

The binding of MDM2 to the *p53* mRNA is regulated by phosphorylation of MDM2 at serine 395 and we wanted to see if the *E2F1* mRNA – MDM2 interaction also involves post-translational modifications of the MDM2 protein. Serine 166 of MDM2 (MDM2-S166) has been linked to cell growth promoting pathways such as AKT/PI3K (47,48) and we tested the importance of this site by substituting serine 166 with an alanine (MDM2-S166A), or we introduced a phosphomimetic aspartic acid (MDM2-S166D). Interestingly, the S166A substitution not only prevented GAr-mediated induction of E2F1 but it also inhibited the expression of E2F1 in a proteasome-independent fashion under translation stress conditions. The S166A mutation had no effect on E2F1 expression under normal conditions, showing that the S166A substitution renders MDM2 dominant negative towards E2F1 under translation stress conditions. The MDM2-S166D instead behaved more like the wild type MDM2 (Figure 3A and Supplementary Figures S4A and S4B). A ^35^S-Met metabolic pulse label confirmed that the MDM2-S166A failed to stimulate translation of the *E2F1* mRNA while MDM2-S166D had a mild stimulatory effect (Figure 3B). We next tested the effect of the MDM2-S166A and MDM2-S166D mutations on the interaction between recombinant MDM2 proteins and the *E2F1* mRNA by expressing the two constructs in H1299 cells followed by mRNA isolation and binding to the respective recombinant proteins. We observed that the MDM2-WT and, MDM2-S166D – *E2F1* mRNA interactions were induced significantly following translation stress, while there was no significant increase in the binding to the MDM2-S166A protein (Figure 3C). As we observed (see above) that translation stress promotes the interaction of the *E2F1 mRNA* to a recombinant MDM2, we wanted to test if MDM2 activated on serine 166 could play a role in folding the *E2F1* mRNA. In support of this, we observed that *in vitro* transcribed *E2F1* mRNA has a poor affinity for the recombinant MDM2 protein but the affinity was enhanced when a phosphomimetic MDM2-S166D was added during *in vitro* transcription. As a comparison, MDM2-166D had no effect on the *p53* mRNA – MDM2 interaction (Figure 3D). This shows that the folding of the *E2F1* mRNA can be regulated to better interact with MDM2 and indicates that MDM2 when activated at serine 166 can act as a chaperone to fold the *E2F1* mRNA.

The *p53* mRNA interacts with the C-terminal RING domain of MDM2 (33) and as we did not expect MDM2 to have more than one RNA binding domain we were surprised to observe that deletion of the RING domain (MDM2-ΔRING) resulted in a constitutive non-regulated binding to the *E2F1* mRNA (Figure 3E and Supplementary Figure S4C). This indicates that MDM2 harbors a cryptic *E2F1* mRNA binding site that is regulated by the RING domain.

We were puzzled by the observation that the MDM2-S166A mutation renders MDM2 dominant negative only under conditions of translation stress and we expected that this site could regulate other aspects of MDM2-dependent synthesis of E2F1, apart from the interaction between MDM2 and the *E2F1* mRNA. To test this, we isolated polysome fractions and by using the MDM2 (4B2), or a serine 166 phosphospecific MDM2 antibody (MDM2 (S166P), we could observe an enrichment of MDM2 and of an MDM2 phosphorylated at S166 on the 80S and polysome fractions following translation stress. This indicates that MDM2 phosphorylated on S166 plays a role in stimulating mRNA translation (Figure 3F). We also observed an approximately 2.5-fold increase in the *E2F1* mRNA levels at the polysome fractions following expression of MDM2 under mRNA translation stress conditions, further underlining an MDM2-dependent induction of *E2F1* mRNA translation (Figure 3G and Supplementary Figure S4D).

### PI3Kδ regulates MDM2 stability and E2F1 synthesis during translation stress

**Figure 4.**
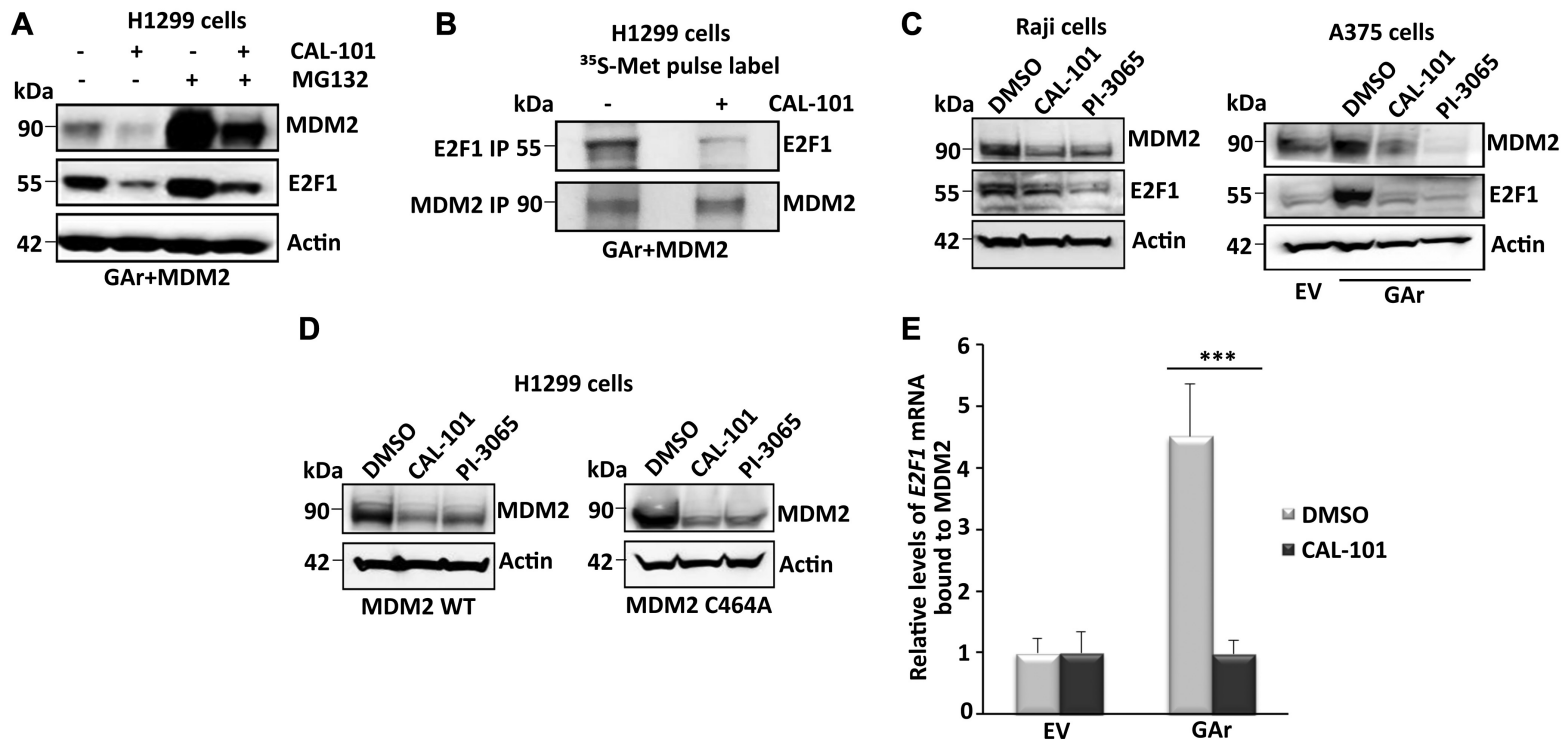
PI3Kδ regulates MDM2 stability and binding to the *E2F1* mRNA. (**A**) WB shows the levels of MDM2 and E2F1 proteins upon treatment with the PI3Kδ inhibitor CAL-101 (5 μM for 8 h) and the proteasome inhibitor MG132 (20 μM for 2 h) in cells expressing the GAr and MDM2. (**B**) Autoradiograph on ^35^S-Met pulse labeling followed by immunoprecipitation using MDM2 or E2F1 antibodies from cells treated, or not, with CAL-101. (**C**) The levels of endogenous E2F1 and MDM2 in the EBV-carrying Burkitt's lymphoma Raji cells (left) following treatment with two PI3Kδ inhibitors CAL-101 and PI-3065 (0.5 μM for 24 hours, respectively). Right panel shows the effect of the PI3Kδ inhibitors on endogenous E2F1 and MDM2 expression in A375 cells expressing the GAr. (**D**) The levels of MDM2 WT and the MDM2-C464A mutant that prevents autoubiquitination, following treatment with two (CAL-101 and PI-3065) PI3Kδ inhibitors (0.5 μM for 24 hours). (**E**) The relative levels of *E2F1* mRNA bound to recombinant MDM2 protein. The RNA was isolated from cells treated with CAL-101 or DMSO. Actin was used a loading control in all WBs and represent one of three independent experiments. Statistical significance was calculated using t tests (***P < 0.001, **P < 0.01 and *P < 0.05).

PI3Kδ is required for translation stress-induced activation of E2F1 and we next set out to test which of the different steps in the induction of E2F1 are regulated by PI3Kδ. PI3Kδ is associated with lymphoid cells but is also expressed in confluent cells like H1299 and the presence of the GAr does not affect P13Kδ levels (Supplementary Figure S5A). Treating cells with the PI3Kδ inhibitor CAL-101 (Idelalisib) resulted in a significant reduction in both MDM2 and E2F1 protein levels that were partially restored when cells were also treated with the proteasome inhibitor MG132 (Figure 4A). To test if the PI3Kδ pathway controls the stability of MDM2 and/or E2F1, or if it affects their respective synthesis, we carried out a metabolic pulse label in cells treated with proteasome inhibitors, with or without CAL-101, followed by IP against MDM2 and E2F1. This showed that treatment with CAL-101 prevents the synthesis of E2F1 but not of MDM2 (Figure 4B), suggesting that one effect of CAL-101 on E2F1 expression is via de-stabilization of MDM2. We also tested the PI3Kδ-inhibitors CAL-101 and PI-3065 on the EBV-carrying Burkitt's lymphoma Raji cells and on A375 cells. The Raji expresses EBNA1 and treatment with either inhibitors suppressed expression of endogenous MDM2 and E2F1. In A375 cells both compounds suppressed the levels of endogenous MDM2 expression and GAr-induced activation of endogenous E2F1 (Figure 4C). Similar, both compounds suppressed the expression of transfected MDM2 in H1299 cells and, interestingly, they also suppressed the expression of an MDM2 that carries a mutation in cysteine 464 (MDM2-C464A) that prevents its E3 ligase activity. This indicates that the effect of PI3Kδ on MDM2 stability is not at the level of MDM2 autoubiquitination (Figure 4D). We next carried out RNA co-IP assays using recombinant MDM2 and total mRNA from cells treated with CAL-101 and we could observe that CAL-101 prevented GAr-induced interaction between MDM2 and the *E2F1* mRNA (Figure 4E). Inhibitors of Akt (Ipatasertib) had a limited effect on the MDM2 – *E2F1* mRNA interaction (Supplementary Figure S5B). Together, these results indicate that the PI3Kδ pathway acts on two levels in regulating MDM2-mediated induction of E2F1: it controls MDM2 stability and the MDM2 - *E2F1* mRNA interaction.

### MDM2-mediated induction of E2F1 induces cell growth pathways, is prevented by the p14Arf and is distinct from its effects on p53

**Figure 5.**
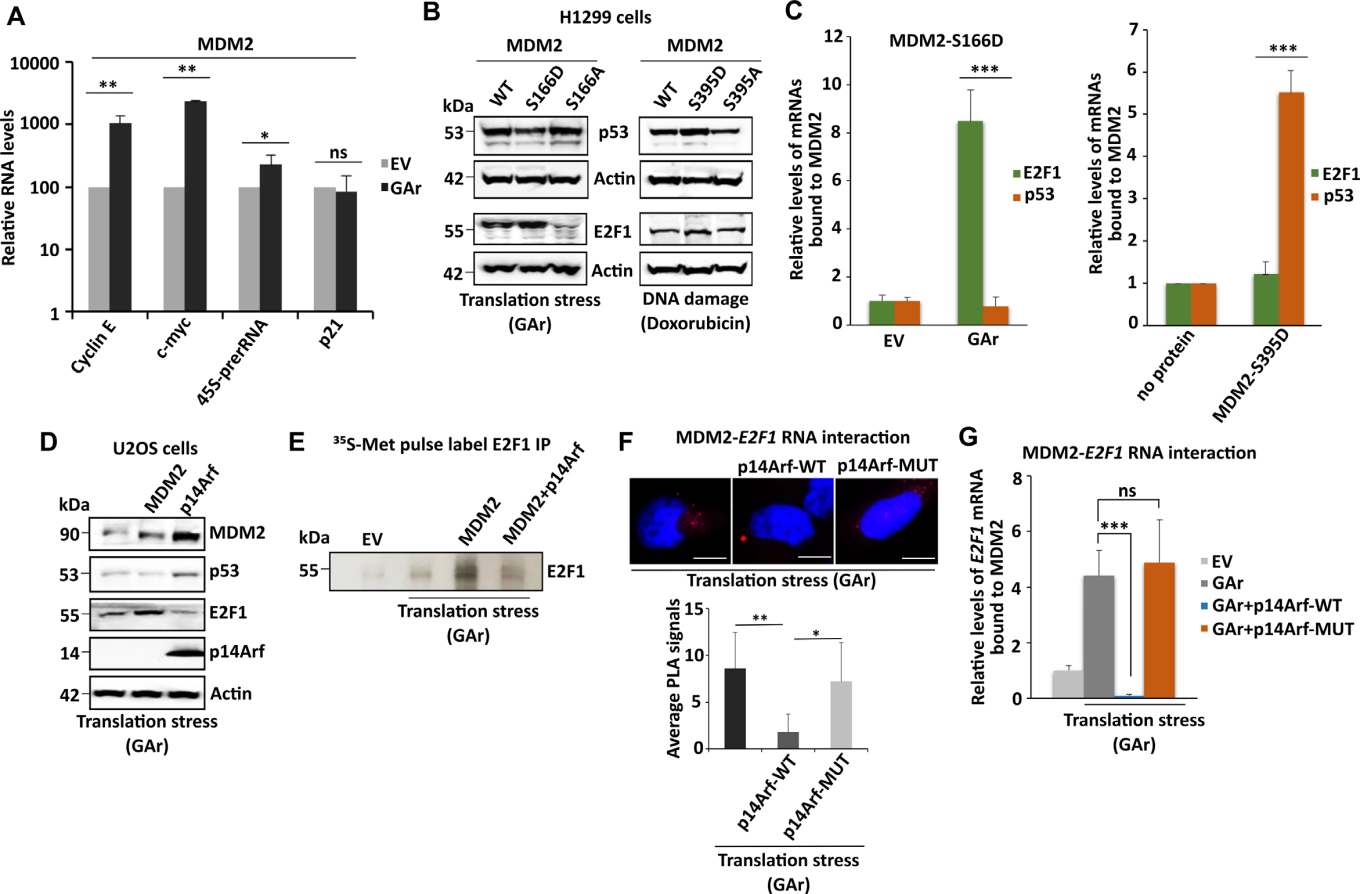
MDM2’s binding to the *E2F1* mRNA is distinct from the binding to the *p53* mRNA and blocked by the tumour suppressor p14Arf. (**A**) mRNA translation stress-mediated induction of E2F1 stimulates growth-promoting genes (cyclin E and c-myc) and ribosome biogenesis (45S pre-rRNA) in cells expressing MDM2. (**B**) WBs show the levels of p53 and E2F1 in cells expressing indicated MDM2 constructs under DNA damage (doxorubicin (1 μM for 4 h)) or mRNA translation stress conditions (GAr). (**C**) The relative levels of *p53* or *E2F1* mRNAs bound to indicated recombinant MDM2 proteins. The left graph shows recombinant MDM2-166D protein binding to indicated mRNAs isolated from cells expressing, or not, the GAr. The right graph shows the binding of indicated mRNAs derived from non-treated cells to a recombinant MDM2 carrying the ATM kinase MDM2-S395D phosphomimetic mutant. No MDM2 protein was used as reference value. (**D**) WBs show p53 and E2F1 levels in p14Arf null U2OS cells expressing p14Arf or MDM2. (**E**) Autoradiograph shows the rate of E2F1 synthesis in the presence of MDM2 and the GAr with, or without, expression of p14Arf. (**F**) PLA shows the interactions between MDM2 and the *E2F1* mRNA in cells expressing p14Arf, or an p14Arf that lacks the MDM2 binding domain (amino acids 2–14) (p14Arf-MUT). The graph below shows the quantification of interactions from three independent experiments. (**G**) The relative amount of *E2F1* mRNA bound to recombinant MDM2. The RNA was isolated from cells expressing indicated constructs. The p14Arf WT protein, but not the p14Arf-MUT, prevented MDM2 from interacting with the E2F1 mRNA. WBs represents one of three independent experiments and actin was used as a loading control. Statistical significance was calculated using t tests (***P < 0.001, **P < 0.01 and *P < 0.05).

MDM2 binds the E2F1 protein and this interaction could potentially inhibit E2F1 activity and to test if MDM2-mediated induction of E2F1 expression following translation stress stimulates downstream cell growth promoting genes we tested MDM2-dependent induction of E2F1 on the expression of cyclin E, c-myc and the 45S pre-ribosomal RNA (pre-rRNA) (49,50). We observed an approximately 10-fold induction of cyclin E, c-myc and 2,5-fold increase in 45S pre-rRNA levels in the presence of MDM2 and during translation stress, as compared to p21^CDKN1A^ that was not affected (Figure 5A). MDM2 is a main regulator of p53 by targeting p53 for degradation under normal conditions or by stimulating its expression during DNA damage. The latter is mediated by the ATM kinase that via phosphorylation of MDM2 serine 395 allows MDM2 to bind the *p53* mRNA and this effect is mimicked by the MDM2-S395D mutation (33). To know if the regulation of p53 and E2F1 synthesis is mutually exclusive, we first expressed the MDM2-S166A or the MDM2-S166D mutants together with p53 or E2F1 during translation or genotoxic stress conditions. The MDM2-S166A prevented E2F1 synthesis during translation stress, as expected, but had no effect on p53 expression under the same conditions. The MDM2-S166D stimulated E2F1 expression while it suppressed p53 levels during the same conditions. Following doxorubicin-induced genotoxic stress (1 μM for 4 h), the MDM2-S395A mutation prevented the induction of p53, as previously shown, while it had little, or no, effect on E2F1 expression. The MDM2-S395D stimulated p53 synthesis during genotoxic stress and showed a limited effect on E2F1 expression (Figure 5B). This indicates that MDM2 stimulates either p53 or E2F1 levels, depending on cellular conditions. To further test this hypothesis and to know if these effects are linked to MDM2’s RNA binding activity, we carried out RNA co-IP using recombinant MDM2-S395D and MDM2-S166D proteins together with total mRNA isolated from H1299 cells expressing the E2F1 or p53 constructs. As expected, the MDM2-S166D bound the *E2F1* mRNA during translation stress but, however, it did not interact with the *p53* mRNA. On the contrary, the ATM phosphomimetic MDM2-S395D protein that binds the *p53* mRNA, did not interact with the *E2F1* mRNA (Figure 5C).

The p14Arf tumour suppressor interacts with MDM2 and prevents its E3 ligase activity towards p53 and we wanted to know if p14Arf also affects MDM2’s activity towards E2F1. When we overexpressed p14Arf in U20S cells (p14Arf-/-) together with E2F1, or p53, we observed an increase in MDM2 and p53 levels, in accordance with a suppression of MDM2’s E3 ligase activity. Importantly, under the same conditions, p14Arf also prevented the expression of E2F1 without affecting *E2F1* mRNA levels (Figure 5D and Supplementary Figure S6). To test if this relates to E2F1 synthesis, we did a metabolic pulse label and we observed that p14Arf indeed prevented MDM2-mediated induction of *E2F1* mRNA translation (Figure 5E). When we expressed the p14Arf and an p14Arf that lacks the N-terminal MDM2-binding domain (amino acids 2–14) (p14Arf-MUT) we observed that the wild type prevented the interaction between MDM2 and the *E2F1* mRNA *in situ* and that the p14Arf-MUT did not (Figure 5F). In line with the *in situ* PLA data, RNA-CoIP data also showed that expression of p14Arf, but not the p14Arf-MUT, prevented recombinant MDM2 from interacting with the *E2F1* mRNA (Figure 5G). These data show that the binding of p14Arf to MDM2 prevents the MDM2 – *E2F1* mRNA interaction. Taken together, MDM2’s activity towards the synthesis of p53 and E2F1 is mediated by two RNA binding domains that are regulated by different cellular pathways and by different post-translational modifications. Furthermore, the p14Arf exhibits a dual tumour suppressor function by preventing MDM2-mediated degradation of p53 and MDM2-mediated synthesis of E2F1.

## DISCUSSION

**Figure 6.**
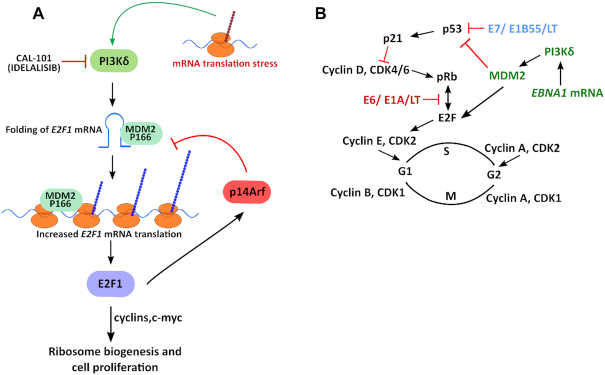
MDM2 regulates E2F1 synthesis during *EBNA1* mRNA-induced translation stress. (**A**) Model illustrating the stress response pathway induced by dysfunctional mRNA translation that via MDM2 induces E2F1 synthesis in a PI3Kδ-dependent fashion which promotes cell cycle progression and increased ribosome biogenesis via c-myc. E2F1 activates the p14Arf tumor suppressor that prevents MDM2-mediated degradation of p53 and MDM2-mediated induction of E2F1 by preventing the MDM2 - *E2F1* mRNA interaction. This illustrates a novel oncogenic activity of MDM2 and a novel tumour suppressor activity of p14Arf. (**B**) Model illustrating how the oncogenic activity of EBNA1-induced mRNA translation stress via PI3Kδ and MDM2 targets the p53 and the retinoblastoma protein (pRb) tumour suppressor pathways. PI3Kδ stabilizes MDM2 and promotes the MDM2 – *E2F1* mRNA interaction that results in an increase in E2F1 protein synthesis. This places the Epstein-Barr virus in the same oncogenic category as Simian virus 40 (SV40), human papilloma virus (HPV) and Adenovirus. However, EBV uses EBNA1-induced mRNA translation stress to target this growth regulatory pathway whereas HPV, Adeno and Simian virus, target the pRb–E2F1 interaction via E6, E1A and Large T antigen (LT) and p53 via E7, E1B55 and LT, respectively. The cell cycle kinase inhibitor p21 (p21^CDKN1A)^) is induced by p53 and prevents phosphorylation of pRb and the release of E2F1.

The self-suppression of *EBNA1* mRNA translation causing E2F1 induction helps to explain two previous independent animal models showing an inverse correlation between EBNA1 protein expression levels and tumour phenotype (10,11). The mRNA translation stress pathway is not unique for EBV-carrying cells and depletion of PI3Kδ suppresses E2F1 expression also in non-EBV-carrying tumour cells. This is consistent with the idea that a high rate of protein synthesis required for cell growth causes translation stress and this feeds back to the E2F1 pathway to stimulate ribosome biogenesis and coordinate cell growth with protein synthesis (Figure 6A). The EBNA1-mediated activation of E2F1 via MDM2 differs from other oncogenic viruses such as Simian, Human papilloma and Adeno that via Large T, E6 and E1A, respectively, compete with E2F1 for the binding to the retinoblastoma protein (pRb). And via Large T, E7 and E1B55, the viruses target p53 (Figure 6B). The EBV-encoded EBNA1 is the first viral oncogene reported to interfere with the pRb and the p53 pathways via its mRNA, and not via the encoded protein. It will be interesting to see if other viruses use a similar concept to stimulate host cell proliferation. The induction of E2F1 requires PI3Kδ and inhibitors of PI3Kδ (Idelalisib) have been tested in the clinic for non-EBV related cancers and it might be worth considering using PI3Kδ inhibitors against EBV-carrying cancers (51). It is interesting to note that patients with gain of function mutations in PI3Kδ and that suffer from the Activated PI3Kδ Syndrome (APDS) have an increase in the transitional B cell population and an increased risk of B cell malignancies (12). The activation of PI3Kδ in EBV-carrying B cells might help to explain the link between EBV and Burkitt's lymphoma and other EBV-linked cancers, including nasopharyngeal carcinoma. On the other hand, the rare cases with mutations that inactivate PI3Kδ would not lead to an induction of E2F1 expression and tumour formation, while causing immune deficiency. Another phenomena linked to APDS is the high susceptibility to herpes virus infections which has been attributed to defects in the T cell population but it is intriguing to note that the EBV-carrying cells have activated PI3Kδ and perhaps part of this phenomena can also be related to a propagation of the infected host cell population.

Another potential therapeutic implication of this study is that treatment of cells with PI3Kδ inhibitors effectively reduces MDM2 levels. Targeting the oncogenic activity of MDM2 is a major focus of the pharmaceutical industry and compounds that prevent its interaction with the p53 protein have been developed (52,53), but reducing MDM2 expression offers an alternative route to target also MDM2’s non p53-dependent oncogenic activity.

The mRNA stress-induced activation of the MDM2 – *E2F1* mRNA pathway involves several steps. Using recombinant MDM2 and *E2F1* mRNA isolated from cells, we observed a four-fold GAr-dependent increase in the MDM2 – *E2F1* mRNA interaction that was prevented by treating cells with the PI3Kδ inhibitor. The RNA extraction excludes RNA-binding proteins, indicating that PI3Kδ, in addition to stabilizing MDM2, promotes the access of the *E2F1* mRNA to MDM2, presumably via the folding of the RNA. This draws parallels with MDM2’s binding to the *p53* mRNA following DNA damage in which the ATM kinase activates HDMX’s RNA chaperone activity to create an *p53* mRNA structure that binds MDM2 (34). We could, however, not observe any effect of MDMX on MDM2-mediated synthesis of E2F1 but we could show that MDM2-S166D folds the *E2F1* mRNA during *in vitro* transcription to form a conformation that promotes binding to MDM2 and that the MDM2-166D has no effect on the MDM2 – *p53* mRNA interaction. There are further similarities between MDM2-mediated induction of p53 and E2F1 synthesis. The binding of MDM2 to the *p53* mRNA requires ATM-mediated phosphorylation of MDM2 at serine 395 which opens an *p53* mRNA binding pocket in MDM2’s C-terminal RING domain. Here we show that the binding to the *E2F1* mRNA is regulated via serine 166 and that deletion of the RING domain instead induces a constitutive binding to MDM2 that is not affected by mRNA translation stress. This indicates that MDM2 harbors at least two cryptic mRNA binding domains. Considering that the effects of binding the *E2F1* or the *p53* mRNAs results in growth promoting, or growth suppressing, pathways, it is rather logic that MDM2’s RNA binding domains are different and regulated independently. This notion is supported by the observation that the MDM2 phosphomimetic mutation MDM2-S395D that stimulates p53 synthesis does not bind the *E2F1* mRNA and has no effect on E2F1 synthesis. Furthermore, the MDM2-S166A mutation that suppresses E2F1 synthesis during translation stress does not affect p53 expression under the same conditions and the phosphomimetic MDM2-S166D which binds the *E2F1* mRNA, does not bind the *p53* mRNA. It is important to keep in mind that the mRNA translation stress pathway exploited by the EBV is also active in non-EBV-infected cells (8) and helps to explain why addition of MDM2 stimulates E2F1 synthesis without the presence of the GAr. However, knocking out MDM2 prevents GAr-mediated induction of E2F1 expression, showing that the GAr is not required for the effect of MDM2 on E2F1 synthesis, while MDM2 is required for the effect of the GAr. Hence, the expression of the EBNA1-encoded GAr further stimulates the translation stress pathway and enhances the MDM2 - *E2F1* mRNA interaction and E2F1 synthesis.

Another consequence of keeping the two mRNA binding sites of MDM2 separate is to allow independent regulation of MDM2’s activity towards p53 and E2F1 by cellular factors. The p14Arf tumour suppressor binds the core domain of MDM2 and prevents MDM2’s E3 ubiquitin ligase activity towards p53. We now show that p14Arf also prevents MDM2 from stimulating E2F1 synthesis by preventing MDM2 from binding the *E2F1* mRNA. Hence, p14Arf exerts a double tumour suppressor activity via its interaction with MDM2. As p14Arf is activated by E2F1 it is possible that MDM2-mediated stimulation of E2F1 under normal conditions results in a negative feedback via the induction of p14Arf. This feedback is abrogated in cancer cells lacking p14Arf, resulting in a constitutive activation of E2F1 in cells experiencing high protein expression and mRNA translation stress. It is notable that MDM2 is overexpressed in approximately 10% of cancers and the fact that it can either have a growth promoting, or suppressive activity, depending on cellular conditions, sheds new light on its role in cancer development.

The MDM2-S166A acts as a dominant negative to prevent E2F1 expression during translation stress but it has little effect on E2F1 synthesis under normal conditions. This suggests that during mRNA translation stress, E2F1 synthesis becomes MDM2-dependent and the incorporation of the MDM2-S166A into the *E2F1* mRNA translation process acts as an inhibitor of synthesis. As serine 166 regulates the MDM2 – *E2F1* mRNA interaction it is plausible that the binding to the *E2F1* mRNA and the stimulation of translation reflect two separate effects which might help explain why MDM2 phosphorylated at serine 166 is detected in the polysome fractions. The notion that RNA binding and translation stimulation are two separate MDM2 activities is further suggested by the observation that MDM2-S166A had no effect on p53 synthesis during translation stress. We have previously shown that MDM2 is at the p53 polysomes during DNA damage and that it plays a key role in ATM-mediated phosphorylation of the nascent p53 protein and it is, thus, possible that a serine 166 activated MDM2 also plays a role at the E2F1 polysomes that extends beyond direct translation stimulation (45). It will be interesting to determine how MDM2 promotes translation of specific mRNAs under different conditions. For example, MDM2 has been implicated to interact with other mRNAs such *XIAP, slug, VEGF* and it will be interesting to know if these interactions are regulated by signaling pathways and by different MDM2 domains (35,54).

MDM2 also binds the E2F1 protein, just like p53, and MDM2 has been reported to stimulate, or inhibit, E2F1 activity. A simple explanation for this apparent controversy might be the levels of MDM2 and the cellular conditions and, indeed, an increase in E2F1 expression is observed at lower MDM2 levels whereas higher amounts results in a decrease in E2F1 expression (Supplementary Figures S2B and S2C). Together, these results suggest a scenario whereby the regulation of MDM2’s interaction with specific mRNAs and with the corresponding proteins in response to cellular conditions and specific signaling pathways, will determine its growth promoting or growth suppressing activity.

## DATA AVAILABILITY

The authors declare that data supporting the findings of this study are available within the article and in supplementary file, or available from the corresponding author upon reasonable request.

## Supplementary Material

gkaa431_Supplemental_File
