# Supplementary material for: MDM2’s dual mRNA binding domains co-ordinate its oncogenic and tumour suppressor activities
Source: Nucleic Acids Res. 2020 May 26;48(12):6775–87. doi: 10.1093/nar/gkaa431 (PMC7337897; doi:10.1093/nar/gkaa431)
Supplement: gkaa431_Supplemental_File [file gkaa431_supplemental_file.pdf]

Supplementary Figure S1

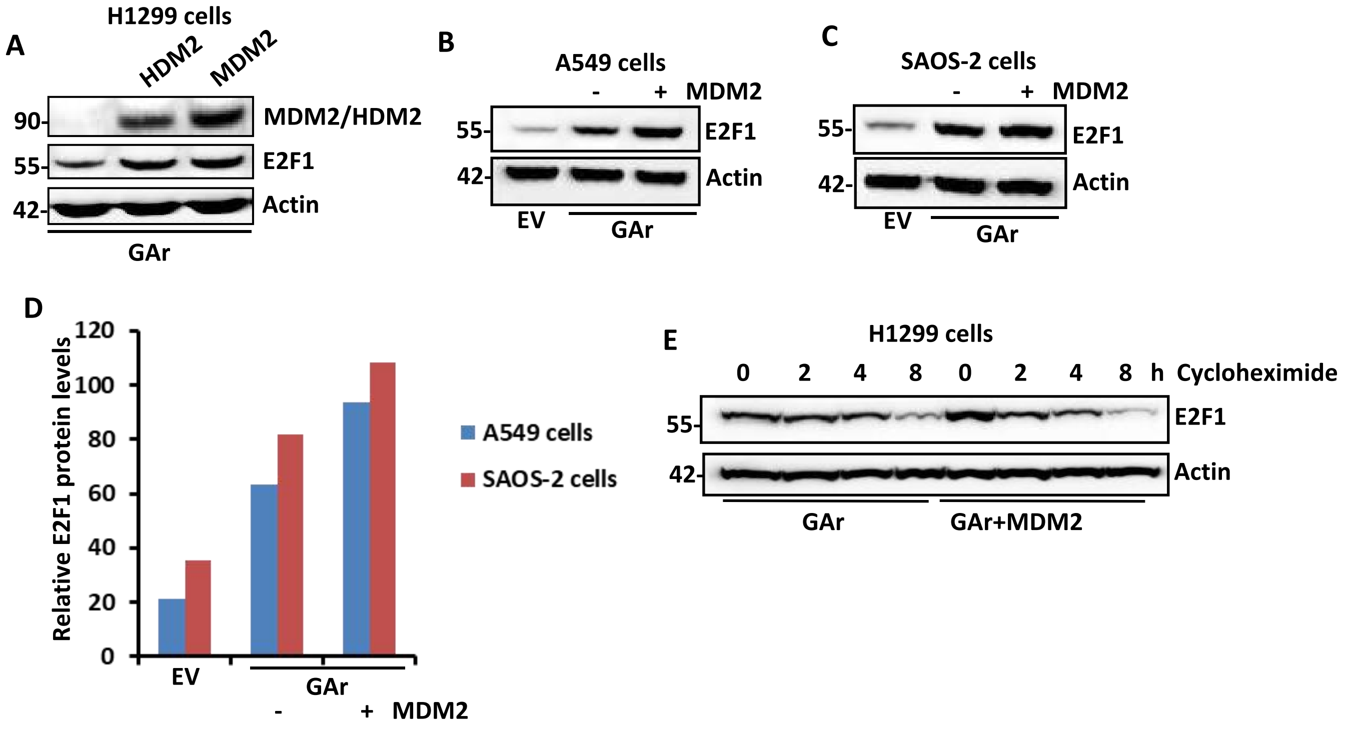

Supplementary Figure S1. MDM2 regulates E2F1 levels during mRNA translation stress. A) Western blot showing E2F1 levels during mRNA translation stress with over-expression of MDM2 (mouse) and HDM2 (human). B and C) Western blots showing E2F1 levels in normal (EV) and mRNA translation stress (GAr) induced cells with over-expression of MDM2 in B) A549 cells (p53 wild type) and c) SAOS-2 cells (p53 null). D) The graph shows the quantification of E2F1 protein levels from B and C. E) Western blot showing the E2F1 levels at indicated time points following Cycloheximide treatment (data relates to Figure 1F graph).

Supplementary Figure S2

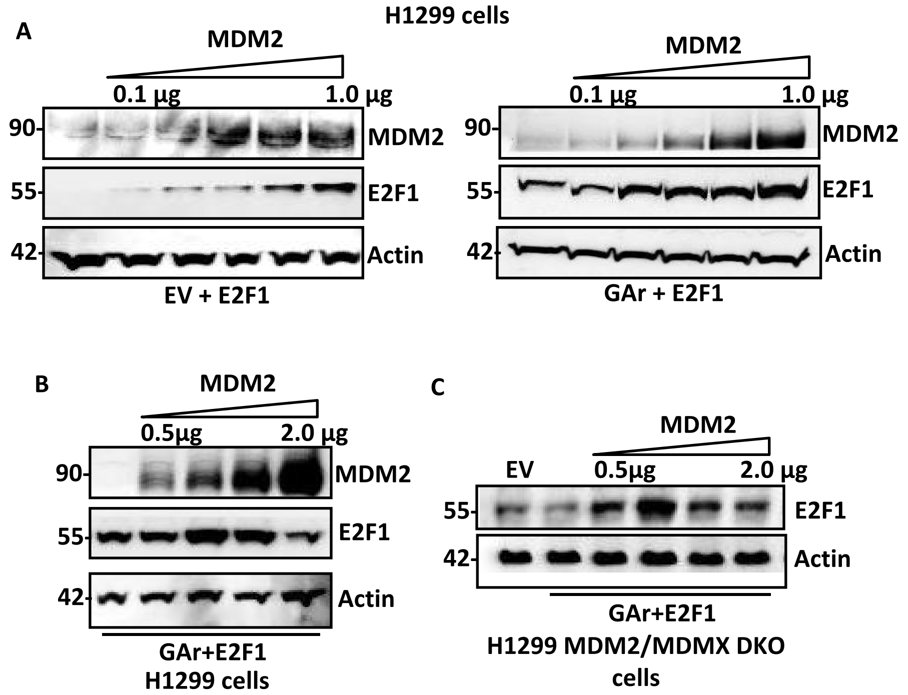

Supplementary Figure S2. A) Western blots showing the dose dependent effect of MDM2 (up to 1 µg) on E2F1 levels in cells expressing the GAr (right) as compared to empty vector (EV) transfected (left). B) and C) Western blots showing the dose dependent effect of MDM2 (up to 2 µg) on E2F1 levels during translation stress in H1299 cells (B) and MDM2/MDMX double KO (DKO) H1299 cells (C). E2F1 levels are reduced at higher concentration of MDM2 (data related to Figure 1c).

Supplementary Figure S3

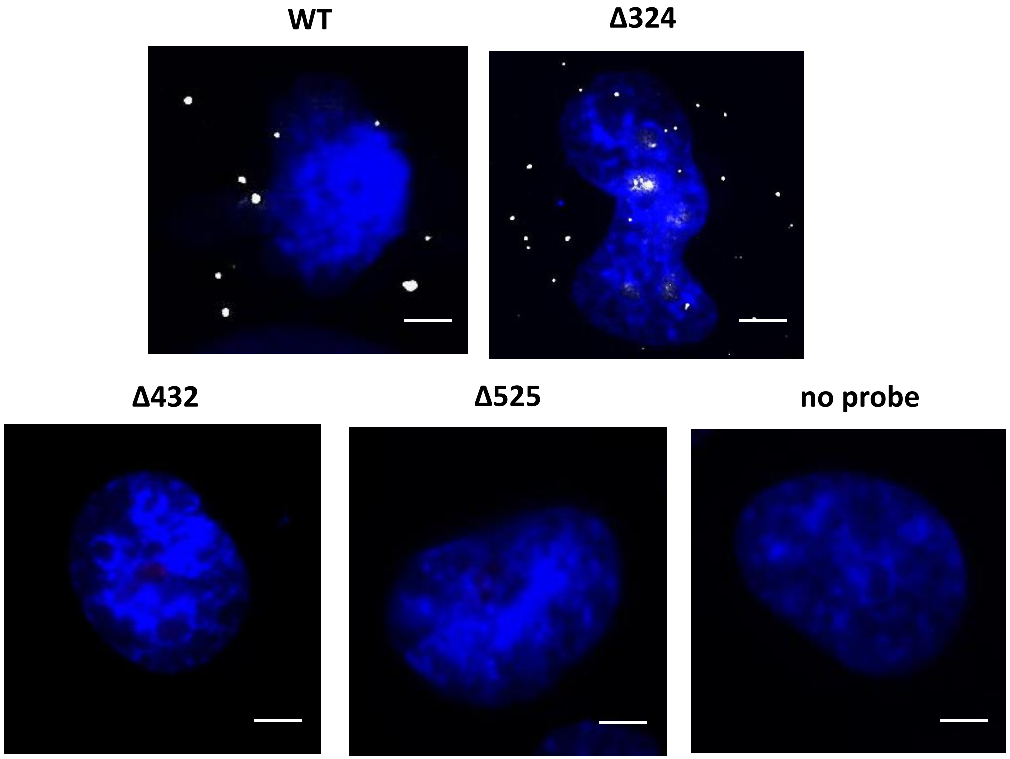

Supplementary Figure S3. Proximity ligation assay (PLA) showing the *in situ* interactions of MDM2 protein - *E2F1* mRNA with indicated E2F1 constructs (E2F1-WT, E2F1-Δ324, E2F1-Δ432 and E2F1-Δ525) under translation stress conditions. PLA without *E2F1* RNA probe (no probe) was used as a negative control. (Data related to quantification shown in Figure 2D). Scale bar 10 µm

Supplementary Figure S4

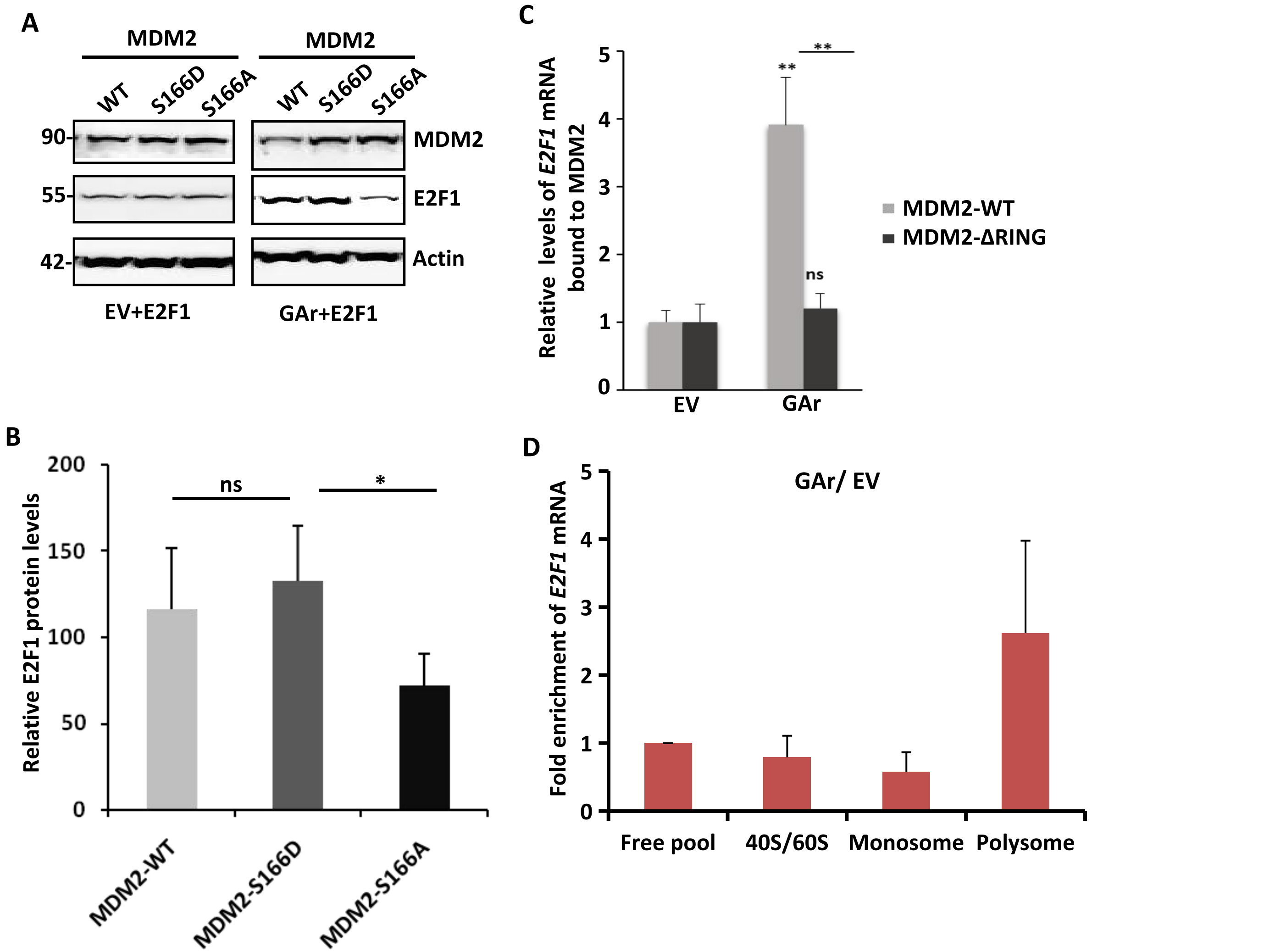

Supplementary Figure S4. A) Western blots showing E2F1 levels upon over-expressing MDM2-WT and phosphomutants (S166D, S166A) under normal (EV) and translation stress conditions (GAr) (data related to Figure 3a). B) Quantification of E2F1 protein levels with over-expression of MDM2-WT, MDM2-S166D, MDM2-S166A C) Graph showing the fold enrichment of *E2F1* mRNA interaction with MDM2-WT and MDM2-ΔRING proteins under normal (EV) and translation stress conditions (GAr) (data related to Figure 3E). D) RT-qPCR analysis of *E2F1* mRNA from corresponding sucrose fractions (Figure 3F), fold enrichment of *E2F1* mRNA in translation stress conditions versus control (EV) was plotted (data related to Figure 3G). Statistical significance was calculated using t-tests (***$p < 0.001$, **$p < 0.01$ and *$p < 0.05$).

Supplementary Figure S5

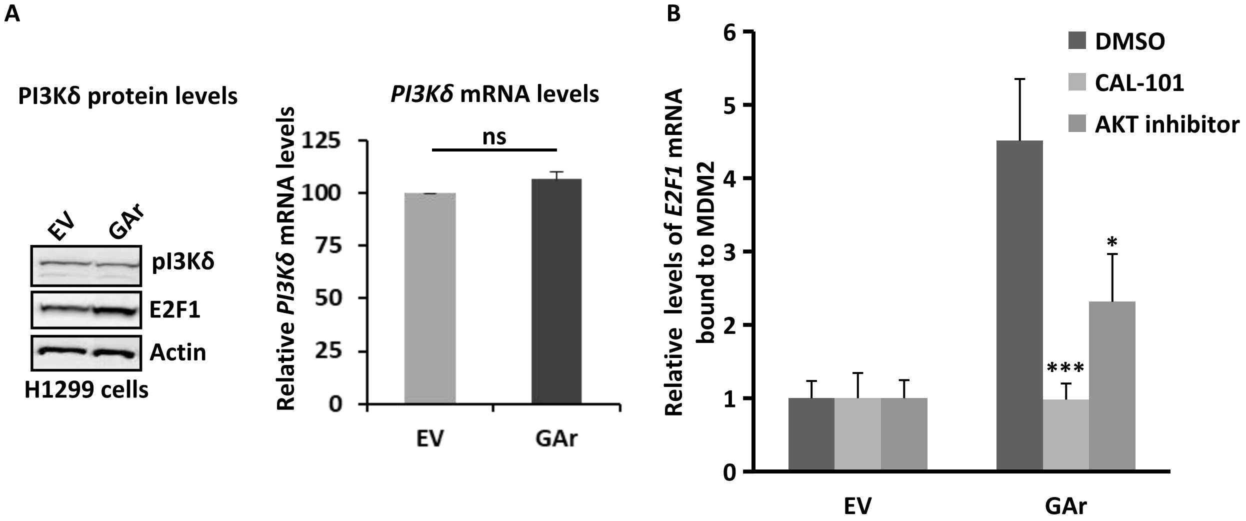

Supplementary Figure S5. A) Left panel; Western blot showing the protein levels of PI3Kδ, right panel; Graph showing the mRNA levels of PI3Kδ, under normal (EV) and translations stress conditions (GAr) in H1299 cells. Values were normalized with actin levels and are representative of three independent experiments. B) Graph showing the fold enrichment of *E2F1* mRNA bound to MDM2 after RNA co-IP with purified MDM2 proteins in cells treated with CAL-101 and AKT inhibitor (Ipatasertib), data related to Figure 4E. Statistical significance was calculated using t-tests (***$p < 0.001$, **$p < 0.01$ and *$p < 0.05$, ns- not significant).

Supplementary Figure S6

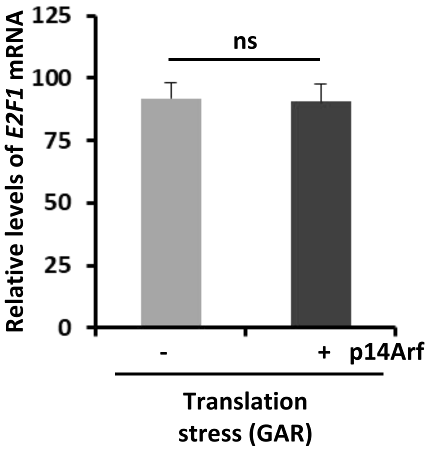

Supplementary Figure S6. Graph showing the mRNA levels of *E2F1*, under translations stress conditions (GAr) in U20S cells with over-expression, or not, of tumour suppressor protein p14Arf. Values were normalized with actin levels and are representative of three independent experiments. Statistical significance was calculated using t-tests (***p < 0.001, **p < 0.01 and *p < 0.05, ns- not significant).

Supplementary Table 1

Oligo	Sequence (5'-3')	Description
E2F1_F	AGGTCACTTCTGAGGAGGAGA	For RT-qPCR
E2F1_R	ACAACAGCGGTTCTTGCTCCA	For RT-qPCR
E2F1 PLA probe	TTCTCCTCCTCAGAAGTGACCTCCTGAAAA	For PLA
c-Myc-F	AACCAGAGTTTCATCTGCGACCCG	For RT-qPCR
c-Myc-R	TTGTGCTGATGTGTGGAGACGTGG	For RT-qPCR
Cyclin E-F	GTTATAAGGGAGACGGGGAG	For RT-qPCR
Cyclin E-R	TGCTCTGCTTCTTACCGCTC	For RT-qPCR
45S pre-rRNA-F	GCTGACACGCTGTCCTCTG	For RT-qPCR
45S pre-rRNA-R	ACGCGCGAGAGAACAGCAG	For RT-qPCR
p21-F	CCTCAAATCGTCCAGCGACCTT	For RT-qPCR
p21-R	CATTGTGGGAGGAGCTGTGAAA	For RT-qPCR
p53-F	TGGGCTTCTTGCATTCTG	For RT-qPCR
p53-R	GCTGTGACTGCTTGTAGATGGC	For RT-qPCR
PI3K_F	ATCCTCGCCATGCGGGATGA	For RT-qPCR
PI3K_R	TGCACCACCAGCTTCATC	For RT-qPCR
β-Actin_F	TCACCCACACTGTGCCCATCTACGA	For RT-qPCR
β -Actin_R	TGAGGTAGTCAGTCAGGTCCCG	For RT-qPCR
MDM2_S166A_F	CTAGAAGGAGAGCAATTGCTGAGACAGAAGAAAATTC	mutagenesis
MDM2_S166A_R	GAATTTTCTTCTGTCTCAGCAATTGCTCTCCTTCTAG	mutagenesis
MDM2_S166D_F	CTAGAAGGAGAGCAATTGATGAGACAGAAGAAAATTC	mutagenesis
MDM2_S166D_R	GAATTTTCTTCTGTCTCATCAATTGCTCTCCTTCTAG	mutagenesis
